# Deaths from Excessive Alcohol Use — United States, 2016–2021

**DOI:** 10.15585/mmwr.mm7308a1

**Published:** 2024-02-29

**Authors:** Marissa B. Esser, Adam Sherk, Yong Liu, Timothy S. Naimi

**Affiliations:** ^1^Division of Population Health, National Center for Chronic Disease Prevention and Health Promotion, CDC; ^2^Canadian Institute for Substance Use Research, Victoria, British Columbia, Canada.

**Summary**

**What is already known about this topic?**

U.S. deaths from causes fully due to excessive alcohol use increased during the past 2 decades.

**What is added by this report?**

Average annual number of deaths from excessive alcohol use, including partially and fully alcohol-attributable conditions, increased approximately 29% from 137,927 during 2016–2017 to 178,307 during 2020–2021, and age-standardized death rates increased from approximately 38 to 48 per 100,000 population. During this time, deaths from excessive drinking among males increased approximately 27%, from 94,362 per year to 119,606, and among females increased approximately 35%, from 43,565 per year to 58,701.

**What are the implications for public health practice?**

Evidence-based alcohol policies (e.g., reducing the number and concentration of places selling alcohol and increasing alcohol taxes) could help reverse increasing alcohol-attributable death rates.

## Abstract

Deaths from causes fully attributable to alcohol use have increased during the past 2 decades in the United States, particularly from 2019 to 2020, concurrent with the onset of the COVID-19 pandemic. However, previous studies of trends have not assessed underlying causes of deaths that are partially attributable to alcohol use, such as injuries or certain types of cancer. CDC’s Alcohol-Related Disease Impact application was used to estimate the average annual number and age-standardized rate of deaths from excessive alcohol use in the United States based on 58 alcohol-related causes of death during three periods (2016–2017, 2018–2019, and 2020–2021). Average annual number of deaths from excessive alcohol use increased 29.3%, from 137,927 during 2016–2017 to 178,307 during 2020–2021; age-standardized alcohol-related death rates increased from 38.1 to 47.6 per 100,000 population. During this time, deaths from excessive alcohol use among males increased 26.8%, from 94,362 per year to 119,606, and among females increased 34.7%, from 43,565 per year to 58,701. Implementation of evidence-based policies that reduce the availability and accessibility of alcohol and increase its price (e.g., policies that reduce the number and concentration of places selling alcohol and increase alcohol taxes) could reduce excessive alcohol use and alcohol-related deaths.

## Introduction

Deaths from causes fully attributable to alcohol use (i.e., 100% alcohol-attributable causes, such as alcoholic liver disease and alcohol use disorder) have increased during the past 2 decades in the United States (*1*); rates were particularly elevated from 2019 to 2020,* concurrent with the onset of the COVID-19 pandemic. In addition, emergency department visit rates associated with acute alcohol use (*2*) and per capita alcohol sales^†^ also increased during this time. Previous studies of trends have not included underlying causes of death that are partially attributable to alcohol (*1*,*3*), such as injuries or certain types of cancer, for which drinking is a substantial risk factor (*4*,*5*). A comprehensive assessment of changes in deaths from excessive alcohol use that includes conditions that are fully and partially attributable to alcohol can guide the rationale for and implementation of effective prevention strategies.

## Methods

### Data Sources and Measures

Total U.S. deaths from alcohol-related conditions during 2016–2021 identified from the National Vital Statistics System were grouped into three periods (2016–2017, 2018–2019, and 2020–2021). Deaths were defined using the underlying cause of death for the 58 alcohol-related conditions^§^ in CDC’s Alcohol-Related Disease Impact (ARDI) application and estimated using ARDI methods.^¶^ For each cause of death, alcohol-attributable fractions were used, reflecting the cause-specific proportion that is due to excessive alcohol use. For the 15 fully alcohol-attributable conditions,** the alcohol-attributable fraction is 1.0. Fully alcohol-attributable conditions include the 100% alcohol-attributable chronic causes as well as the 100% alcohol-attributable acute causes (i.e., alcohol poisonings that are a subset of deaths in the alcohol-related poisonings category and deaths from suicide by exposure to alcohol that are a subset of the suicide category). Partially alcohol-attributable conditions are those that are caused by alcohol use or other factors, and alcohol-attributable fractions are applied to calculate the deaths from alcohol use. For most of the partially alcohol-related chronic conditions, population-attributable fractions were estimated using relative risks from published meta-analyses and adjusted prevalence estimates of low, medium, and high average daily alcohol use among U.S. adults. Prevalence estimates were obtained from the Behavioral Risk Factor Surveillance System^††^ and adjusted using alcohol per capita sales information to account for underreporting of self-reported drinking (*6*). 

Alcohol-attributable fractions for acute causes (e.g., injuries) were determined mostly from a recent meta-analysis that generally measured the proportion of decedents who had a blood alcohol concentration (BAC) ≥0.10% (*7*). Alcohol-attributable fractions for motor vehicle crashes and other road vehicle crash deaths were obtained from the Fatality Analysis Reporting System, based on the proportion of crash deaths that involved a decedent with BAC ≥0.08%.^§§^

Deaths from excessive alcohol use (as opposed to deaths from any level of drinking) includes all decedents whose deaths were attributed to conditions that are fully caused by alcohol use, alcohol-related acute causes of death that involved binge drinking, and alcohol-related chronic conditions that involved medium or high average daily levels of alcohol use. For the chronic causes of death estimated using cause-specific population-attributable fractions by sex, the relative risks for death at medium daily average drinking levels (females: >1 to ≤2 drinks, males: >2 to ≤4 drinks) and high daily average drinking levels (females: >2 drinks, males: >4 drinks) were relative to the risks at low daily average drinking levels (females: >0 to ≤1 drink, males: >0 to ≤2 drinks).

### Data Analyses

The average annual number of deaths resulting from excessive alcohol use during three 2-year periods, percentage change in numbers of deaths,^¶¶^ and death rates were calculated overall and by sex and cause of death category. The number of sex-stratified deaths from excessive drinking was also assessed by age group. In general, deaths from chronic conditions were calculated among decedents aged ≥20 years, and deaths from acute causes were calculated among decedents aged ≥15 years. Younger children whose deaths resulted from someone else’s drinking (e.g., as passengers in motor vehicle crashes) were also included for several causes of death. Death rates (deaths per 100,000 population) were calculated based on midyear postcensal population estimates and age-standardized to the 2000 U.S. Census Bureau standard population. Nonoverlapping 95% CIs were considered significantly different. Analyses were conducted using SAS software (version 9.4; SAS Institute). This activity was reviewed by CDC, deemed not research, and was conducted consistent with applicable federal law and CDC policy.***

## Results

Average annual deaths from excessive alcohol use in the United States increased 5.3%, from 137,927 during 2016–2017 to 145,253 during 2018–2019; these deaths then increased more sharply (22.8%) from 2018–2019 to 178,307 during 2020–2021, for an overall 29.3% increase from 2016–2017 to 2020–2021 (Table 1). Age-standardized death rates increased from 38.1 per 100,000 population during 2016–2017 to 39.1 during 2018–2019 to 47.6 during 2020–2021. Approximately two thirds of these deaths resulted from chronic causes during each period: alcohol-attributable death rates from chronic causes increased from 23.2 per 100,000 population to 24.3 to 29.4 during the respective analysis periods. During 2020–2021, fully alcohol-attributable causes^†††^ accounted for 51,665 deaths (29.0% of all alcohol-attributable deaths), a 46.2% increase compared with the 35,344 deaths that occurred during 2016–2017. During 2020–2021, partially alcohol-attributable causes accounted for 126,642 deaths (71.0% of all alcohol-attributable deaths), a 23.5% increase compared with the 102,583 partially alcohol-attributable deaths that occurred during 2016–2017.

**TABLE 1:** Average annual number of deaths from excessive alcohol use, cause of death, and age-standardized death rates, by period — United States, 2016–2021

Cause of death	Average annual no. of deaths from excessive alcohol use			% Change in average annual no. of deaths from excessive alcohol use			Age-standardized deaths per 100,000 population (95% CI)		
	2016–2017	2018–2019	2020–2021	2018–2019 vs. 2016–2017	2020–2021 vs. 2018–2019	2020–2021 vs. 2016–2017	2016–2017	2018–2019	2020–2021
All causes*	**137,927**	**145,253**	**178,307**	**5.3**	**22.8**	**29.3**	**38.1 (37.9–38.3)**	**39.1 (38.9–39.3)^†^**	**47.6 (47.4–47.8)^†,§^**
**Chronic cause**									
**All chronic causes**	**88,587**	**95,462**	**117,245**	**7.8**	**22.8**	**32.4**	**23.2 (23.1–23.4)**	**24.3 (24.1–24.5)^†^**	**29.4 (29.3–29.6)^†,§^**
100% alcohol-attributable (chronic)^¶^	32,937	35,819	48,972	8.8	36.7	48.7	9.0 (8.9–9.1)	9.6 (9.3–9.7)^†^	13.2 (13.1–13.3)^†,§^
Cancer**	16,123	16,686	17,072	3.5	2.3	5.9	4.1 (4.0–4.2)	4.1 (4.0–4.1)	4.0 (4.0–4.1)
Heart disease and stroke^††^	27,952	30,814	37,317	10.2	21.1	33.5	7.2 (7.1–7.2)	7.5 (7.5–7.6)^†^	8.8 (8.7–8.8)^†,§^
Liver, gallbladder, and pancreas^§§^	10,673	11,178	12,719	4.7	13.8	19.2	2.8 (2.7–2.8)	2.8 (2.7–2.8)	3.1 (3.1–3.2)^†,§^
Other chronic cause^¶¶^	902	965	1,165	7.0	20.7	29.2	0.3 (0.2–0.3)	0.3 (0.2–0.3)	0.3 (0.3–0.4)
**Acute cause**									
**All acute causes**	**49,340**	**49,791**	**61,063**	**0.9**	**22.6**	**23.8**	**14.9** **(14.8–15.0)**	**14.8** **(14.7–15.0)**	**18.2** **(18.0–18.3)^†,§^**
Alcohol-related poisoning***	14,944	15,400	21,806	3.1	41.6	45.9	4.6 (4.5–4.7)	4.7 (4.6–4.8)	6.6 (6.5–6.7)^†,§^
Motor vehicle traffic crash	13,009	12,579	15,055	–3.3	19.7	15.7	4.0 (3.9–4.0)	3.8 (3.7–3.9)	4.5 (4.5–4.6)^†,§^
Suicide^†††^	9,608	9,974	9,801	3.8	–1.7	2.0	2.9 (2.8–2.9)	2.9 (2.9–3.0)	2.9 (2.8–2.9)
Other acute cause^§§§^	11,779	11,838	14,400	0.5	21.6	22.3	3.5 (3.4–3.5)	3.4 (3.4–3.5)	4.2 (4.1–4.2)^†,§^

* Includes 58 causes of death related to alcohol use. Deaths from excessive alcohol use includes all decedents whose deaths were attributed to conditions that were fully caused by alcohol use, alcohol-related acute causes of death that involved binge drinking, and alcohol-related chronic conditions that involved medium (females: >1 to ≤2 drinks, males: >2 to ≤4 drinks) or high (females: >2 drinks, males: >4 drinks) daily average drinking levels.

^†^ Nonoverlapping 95% CIs of age-standardized death rates compared with 2016–2017.

^§^ Nonoverlapping 95% CIs of age-standardized death rates compared with 2018–2019.

^¶^ The 100% alcohol-attributable chronic causes of death included alcohol abuse, alcohol cardiomyopathy, alcohol dependence syndrome, alcohol polyneuropathy, alcohol-induced acute pancreatitis, alcohol-induced chronic pancreatitis, alcoholic gastritis, alcoholic liver disease, alcoholic myopathy, and alcoholic psychosis.

** Cancer deaths from excessive alcohol use were estimated for deaths from breast cancer (females only), colorectal cancer, esophageal cancer (for the proportion due to squamous cell carcinoma only, based on the Surveillance, Epidemiology, and End Results Program data in 17 states), laryngeal cancer, liver cancer, oral cavity and pharyngeal cancer, pancreatic cancer, prostate cancer (males only), and stomach cancer. Deaths from pancreatic and stomach cancers were calculated among people consuming high daily average levels of alcohol only (females: >2 drinks, males: >4 drinks).

^††^ Deaths from excessive alcohol use from heart disease and stroke were estimated for deaths from atrial fibrillation, coronary heart disease, hemorrhagic stroke, hypertension, and ischemic stroke.

^§§^ Deaths from excessive alcohol use were estimated for deaths from conditions of the gallbladder, liver, and pancreas, including those from acute pancreatitis, chronic pancreatitis, esophageal varices, gallbladder disease, gastroesophageal hemorrhage, portal hypertension, and unspecified liver cirrhosis.

^¶¶^ Deaths from excessive alcohol use were estimated for deaths from other chronic conditions including chronic hepatitis; infant deaths due to low birthweight, preterm birth, and small for gestational age; pneumonia; and seizure disorder, seizures, and unprovoked epilepsy.

*** Deaths from alcohol-related poisonings included those from alcohol poisoning (100% attributable to alcohol) and the portion of deaths from poisonings that involved another substance (e.g., drug overdoses) in addition to a high blood alcohol concentration (≥0.10%).

^†††^ Deaths from excessive alcohol use from suicide included those from suicide by exposure to alcohol (100% attributable to alcohol) and a portion of deaths from suicide based on the alcohol-attributable fraction of 0.21.

^§§§^ Deaths from excessive alcohol use from other acute conditions included the cause-specific portion of deaths for air-space transport, aspiration, child maltreatment, drowning, fall injuries, fire injuries, firearm injuries, homicide, hypothermia, motor vehicle nontraffic crashes, occupational and machine injuries, other road vehicle crashes, and water transport.

### Increases Among Males and Females

The average annual number of deaths from excessive alcohol use among males increased by 25,244 (26.8%), from 94,362 deaths during 2016–2017 to 119,606 during 2020–2021 (Table 2). Age-standardized death rates among males increased from 54.8 per 100,000 population during 2016–2017 to 55.9 during 2018–2019, and to 66.9 during 2020–2021. During each period, among all excessive alcohol use cause of death categories, death rates among males were highest from 100% alcohol-attributable chronic conditions.

**TABLE 2:** Average annual number of deaths from excessive alcohol use, cause of death, and age-standardized death rates, by period and sex — United States, 2016–2021

Cause of death/Sex	Average annual no. of deaths from excessive alcohol use			% Change in average annual no. of deaths from excessive alcohol use			Deaths per 100,000 population (95% CI)		
	2016–2017	2018–2019	2020–2021	2018–2019 vs. 2016–2017	2020–2021 vs. 2018–2019	2020–2021 vs. 2016–2017	2016–2017	2018–2019	2020–2021
**Male**									
All causes*	**94,362**	**98,637**	**119,606**	**4.5**	**21.3**	**26.8**	**54.8 (54.5–55.2)**	**55.9 (55.6–56.3)^†^**	**66.9 (66.5–67.3)^†,§^**
**Chronic cause**									
**All chronic causes**	**57,791**	**61,746**	**73,921**	**6.8**	**19.7**	**27.9**	**32.4 (32.2–32.7)**	**33.6 (33.3–33.9)^†^**	**39.4 (39.1–39.7)^†,§^**
100% alcohol-attributable (chronic)^¶^	23,753	25,600	34,811	7.8	36.0	46.6	13.4 (13.3–13.6)	14.3 (14.1–14.4)^†^	19.2 (19.0–19.5)^†,§^
Cancer**	12,367	12,815	12,647	3.6	–1.3	2.3	6.8 (6.7–6.9)	6.8 (6.7–6.9)	6.4 (6.3–6.5)^†,§^
Heart disease and stroke^††^	14,985	16,378	18,663	9.3	14.0	24.5	8.4 (8.3–8.6)	8.8 (8.7–8.9)^†^	9.6 (9.5–9.7)^†,§^
Liver, gallbladder, and pancreas^§§^	6,021	6,229	6,949	3.5	11.6	15.4	3.3 (3.3–3.4)	3.4 (3.3–3.4)	3.6 (3.6–3.7)^†,§^
Other chronic cause^¶¶^	664	724	851	9.0	17.5	28.2	0.4 (0.4–0.4)	0.4 (0.4–0.4)	0.5 (0.4–0.5)
**Acute cause**									
**All acute causes**	**36,571**	**36,891**	**45,685**	**0.9**	**23.8**	**24.9**	**22.4 (22.2–22.6)**	**22.3 (22.1–22.5)**	**27.5 (27.2–27.8)^†,§^**
Alcohol-related poisoning***	10,315	10,789	15,557	4.6	44.2	50.8	6.4 (6.3–6.5)	6.7 (6.5–6.8)	9.5 (9.4–9.7)^†,§^
Motor vehicle traffic crash	9,910	9,450	11,364	–4.6	20.3	14.7	6.1 (6.0–6.2)	5.7 (5.6–5.9)^†^	6.9 (6.7–7.0)^†,§^
Suicide^†††^	7,465	7,815	7,812	4.7	—^§§§^	4.6	4.5 (4.4–4.6)	4.7 (4.6–4.8)	4.6 (4.5–4.7)
Other acute cause^¶¶¶^	8,881	8,836	10,953	–0.5	24.0	23.3	5.4 (5.3–5.5)	5.3 (5.2–5.4)	6.5 (6.4–6.6)^†,§^
**Female**									
All causes*	**43,565**	**46,616**	**58,701**	**7.0**	**25.9**	**34.7**	**22.7 (22.5–22.9)**	**23.6 (23.3–23.8)^†^**	**29.4 (29.1–29.6)^†,§^**
**Chronic cause**									
**All chronic causes**	**30,796**	**33,717**	**43,324**	**9.5**	**28.5**	**40.7**	**15.1 (15.0–15.3)**	**16.0 (15.9–16.2)^†^**	**20.3 (20.1–20.5)^†,§^**
100% alcohol-attributable (chronic)^¶^	9,184	10,220	14,161	11.3	38.6	54.2	4.9 (4.8–5.0)	5.4 (5.3–5.5)^†^	7.6 (7.4–7.7)^†,§^
Cancer**	3,756	3,871	4,426	3.1	14.3	17.8	1.8 (1.7–1.9)	1.8 (1.7–1.8)	2.0 (1.9–2.0)^§^
Heart disease and stroke^††^	12,967	14,436	18,653	11.3	29.2	43.8	6.0 (5.9–6.1)	6.4 (6.3–6.5)^†^	8.0 (7.9–8.1)^†,§^
Liver, gallbladder, and pancreas^§§^	4,652	4,949	5,770	6.4	16.6	24.0	2.2 (2.2–2.3)	2.3 (2.2–2.4)	2.6 (2.6–2.7)^†,§^
Other chronic cause^¶¶^	238	240	314	0.8	30.8	31.9	0.1 (0.1–0.2)	0.1 (0.1–0.2)	0.2 (0.2–0.2)
**Acute cause**									
**All acute causes**	**12,769**	**12,900**	**15,378**	**1.0**	**19.2**	**20.4**	**7.5 (7.4–7.7)**	**7.5 (7.4–7.7)**	**9.0 (8.9–9.2)^†,§^**
Alcohol-related poisoning***	4,629	4,610	6,249	–0.4	35.6	35.0	2.8 (2.7–2.9)	2.8 (2.7–2.9)	3.8 (3.7–3.9)^†,§^
Motor vehicle traffic crash	3,098	3,128	3,691	1.0	18.0	19.1	1.9 (1.8–2.0)	1.9 (1.8–2.0)	2.2 (2.2–2.3)^†,§^
Suicide^†††^	2,143	2,159	1,990	0.7	–7.8	–7.1	1.3 (1.2–1.3)	1.3 (1.2–1.3)	1.2 (1.1–1.2)
Other acute cause^¶¶¶^	2,898	3,002	3,448	3.6	14.9	19.0	1.6 (1.5–1.7)	1.6 (1.6–1.7)	1.9 (1.8–1.9)^†,§^

* Includes 58 causes of death related to alcohol use. Deaths from excessive alcohol use includes all decedents whose deaths were attributed to conditions that were fully caused by alcohol use, alcohol-related acute causes of death that involved binge drinking, and alcohol-related chronic conditions that involved medium (females: >1 to ≤2 drinks, males: >2 to ≤4 drinks) or high (females: >2 drinks, males: >4 drinks) daily average drinking levels.

^†^ Nonoverlapping 95% CIs of age-standardized death rates compared with 2016–2017.

^§^ Nonoverlapping 95% CIs of age-standardized death rates compared with 2018–2019.

^¶^ The 100% alcohol-attributable chronic causes of death included alcohol abuse, alcohol cardiomyopathy, alcohol dependence syndrome, alcohol polyneuropathy, alcohol-induced acute pancreatitis, and alcohol-induced chronic pancreatitis, alcoholic gastritis, alcoholic liver disease, alcoholic myopathy, and alcoholic psychosis.

** Cancer deaths from excessive alcohol use were estimated for deaths from breast cancer (females only), colorectal cancer, esophageal cancer (for the proportion due to squamous cell carcinoma only, based on the Surveillance, Epidemiology, and End Results Program data in 17 states), laryngeal cancer, liver cancer, oral cavity and pharyngeal cancer, pancreatic cancer, prostate cancer (males only), and stomach cancer. Deaths from pancreatic and stomach cancers were calculated among people consuming high daily average levels of alcohol only (females: >2 drinks, males: >4 drinks).

^††^ Deaths from excessive alcohol use from heart disease and stroke were estimated for deaths from atrial fibrillation, coronary heart disease, hemorrhagic stroke, hypertension, and ischemic stroke.

^§§^ Deaths from excessive alcohol use were estimated for deaths from conditions of the gallbladder, liver, and pancreas including those from acute pancreatitis, chronic pancreatitis, esophageal varices, gallbladder disease, gastroesophageal hemorrhage, portal hypertension, and unspecified liver cirrhosis.

^¶¶^ Deaths from excessive alcohol use were estimated for deaths from other chronic conditions including chronic hepatitis; infant deaths due to low birth weight, preterm birth, and small for gestational age; pneumonia; and seizure disorder, seizures, and unprovoked epilepsy.

*** Deaths from alcohol-related poisonings included those from alcohol poisoning (100% attributable to alcohol) and the portion of deaths from poisonings that involved another substance (e.g., drug overdoses) in addition to a high blood alcohol concentration (≥0.10%).

^†††^ Deaths from excessive alcohol use from suicide included those from suicide by exposure to alcohol (100% attributable to alcohol) and a portion of deaths from suicide based on the alcohol-attributable fraction of 0.21.

^§§§^ No change in percentage of average annual deaths.

^¶¶¶^ Deaths from excessive alcohol use from other acute conditions included the cause-specific portion of deaths for air-space transport, aspiration, child maltreatment, drowning, fall injuries, fire injuries, firearm injuries, homicide, hypothermia, motor vehicle nontraffic crashes, occupational and machine injuries, other road vehicle crashes, and water transport.

Among females, the average annual number of deaths from excessive alcohol use increased by 15,136 (34.7%), from 43,565 during 2016–2017, to 58,701 during 2020–2021. Age-standardized alcohol-attributable death rates among females increased from 22.7 per 100,000 population during 2016–2017 to 23.6 during 2018–2019, and to 29.4 during 2020–2021. Death rates among females were highest from heart disease and stroke during each period. Among both males and females, alcohol-attributable death rates increased for most cause of death categories. The average number of sex-specific alcohol-attributable deaths increased among all age groups from 2016–2017 to 2020–2021(Figure).

**FIGURE:**
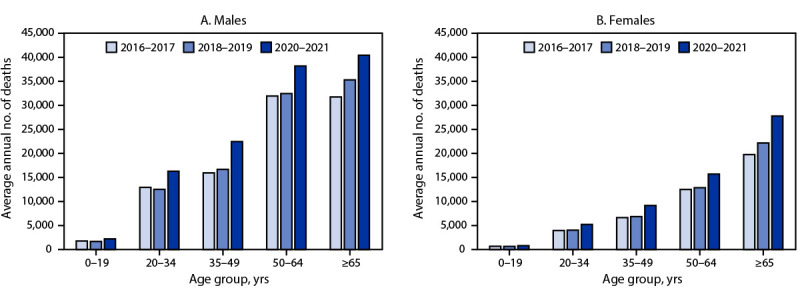
Average annual number of deaths from excessive alcohol use,* by age group and period among males (A) and females (B) — United States, 2016–2021 * Deaths from excessive alcohol use includes all decedents whose deaths were attributed to conditions that were fully caused by alcohol use, alcohol-related acute causes of death that involved binge drinking, and alcohol-related chronic conditions that involved medium (females: >1 to ≤2 drinks, males: >2 to ≤4 drinks) or high (females: >2 drinks, males: >4 drinks) daily average drinking levels.

## Discussion

From 2016–2017 to 2020–2021, the average annual number of U.S. deaths from excessive alcohol use increased by more than 40,000 (29%), from approximately 138,000 per year (2016–2017) to 178,000 per year (2020–2021). This increase translates to an average of approximately 488 deaths each day from excessive drinking during 2020–2021. From 2016–2017 to 2020–2021, the average annual number of deaths from excessive alcohol use increased by more than 25,000 among males and more than 15,000 among females; however, the percentage increase in the number of deaths during this time was larger for females (approximately 35% increase) than for males (approximately 27%). These findings are consistent with another recent study that found a larger increase in fully alcohol-attributable death rates among females compared with males (*8*).

Increases in deaths from excessive alcohol use during the study period occurred among all age groups. A recent study found that one in eight total deaths among U.S. adults aged 20–64 years during 2015–2019 resulted from excessive alcohol use (*9*). Because of the increases in these deaths during 2020–2021, including among adults in the same age group, excessive alcohol use could account for an even higher proportion of total deaths during that 2-year period. In addition, data from Monitoring the Future, an ongoing study of the behaviors, attitudes, and values of U.S. residents from adolescence through adulthood, showed that the prevalence of binge drinking among adults aged 35–50 years was higher in 2022 than in any other year during the past decade^§§§^; this increase could contribute to future increases in alcohol-attributable deaths. In this study, fewer than one third of deaths from excessive alcohol use were from fully alcohol-attributable causes, highlighting the importance of also assessing partially alcohol-attributable causes to better understand the harms from excessive drinking, including binge drinking.

The nearly 23% increase in the deaths from excessive alcohol use that occurred from 2018–2019 to 2020–2021 was approximately four times as high as the previous 5% increase that occurred from 2016–2017 to 2018–2019. Increases in the availability of alcohol in many states might have contributed to this disproportionate increase (*10*). During the peak of the COVID-19 pandemic in 2020–2021, policies were widely implemented to expand alcohol carryout and delivery to homes, and places that sold alcohol for off-premise consumption (e.g., liquor stores) were deemed as essential businesses in many states (and remained open during lockdowns).^¶¶¶^ General delays in seeking medical attention, including avoidance of emergency departments**** for alcohol-related conditions^††††^; stress, loneliness, and social isolation; and mental health conditions might also have contributed to the increase in deaths from excessive alcohol use during the COVID-19 pandemic.

### Limitations

The findings in this report are subject to at least two limitations. First, population-attributable fractions were calculated based on data including only persons who currently drank alcohol. Because some persons who formerly drank alcohol might also die from alcohol-related causes, population-attributable fractions might underestimate alcohol-attributable deaths. Second, several conditions (e.g., HIV/AIDS and tuberculosis) for which excessive alcohol use is a substantial risk factor were not included because relative risk estimates relevant to the U.S. population were not available for calculating the portion of these deaths attributable to drinking alcohol, further contributing to conservative death estimates in this report.

### Implications for Public Health Practice

States and communities can discourage excessive alcohol use and reverse recent increases in alcohol-attributable deaths by implementing comprehensive strategies, including evidence-based alcohol policies that reduce the availability and accessibility of alcohol and increase its price (e.g., policies that reduce the number and concentration of places selling alcohol and increase alcohol taxes).^§§§§^ Also, CDC’s electronic screening and brief intervention^¶¶¶¶^ can be used in primary and acute care, or nonclinical, settings to allow adults to check their alcohol use, receive personalized feedback, and create a plan for drinking less alcohol. Integration of screening and brief intervention into routine clinical services***** for adults and mass media communications campaigns^†††††^ to support people in drinking less can also help. Increased use of these strategies, particularly effective alcohol policies, could help reduce excessive alcohol use and related deaths among persons who drink and also reduce harms to persons who are affected by others’ alcohol use (e.g., child and adult relatives, friends, and strangers).

## Footnotes

*
https://www.cdc.gov/nchs/products/databriefs/db448.htm

†
https://www.niaaa.nih.gov/publications/surveillance-reports/surveillance120

§
https://www.cdc.gov/alcohol/ardi/alcohol-related-icd-codes.html

¶
https://www.cdc.gov/alcohol/ardi/methods.html

** Deaths from causes fully attributable to alcohol use (i.e., 100% alcohol-attributable causes) include alcohol abuse, alcohol cardiomyopathy, alcohol dependence syndrome, alcohol poisoning, alcohol polyneuropathy, alcohol-induced acute pancreatitis, alcohol-induced chronic pancreatitis, alcoholic gastritis, alcoholic liver disease, alcoholic myopathy, alcoholic psychosis, degeneration of the nervous system due to alcohol use, fetal alcohol syndrome, fetus and newborn issues caused by maternal alcohol use, and suicide by exposure to alcohol.

†† Daily average alcohol use prevalence estimates were from the Behavioral Risk Factor Surveillance System (https://www.cdc.gov/brfss/data_documentation/index.htm), aligning with the respective years of the death data for the three periods in this study, and used in population attributable fraction calculations. Prevalence estimates of daily average alcohol use were calculated for three levels, including low (females: >0 to ≤1 drink, males: >0 to ≤2 drinks), medium (females: >1 to ≤2 drinks, males: >2 to ≤4 drinks), and high (females: >2 drinks, males: >4 drinks), unless the source of the relative risk estimates specified otherwise. For the three periods in this study, the categorical relative risks were calculated to correspond with the median of the alcohol use distribution for each drinking level.

§§
https://www.nhtsa.gov/research-data/fatality-analysis-reporting-system-fars

¶¶ The percentage represents the equation {[(estimated average annual number of deaths from excessive alcohol use in more recent period − estimated average annual number of deaths from excessive alcohol use in earlier period) / estimated average annual number of deaths from excessive alcohol use in earlier period] x 100}.

*** 45 C.F.R. part 46.102(l)(2), 21 C.F.R. part 56; 42 U.S.C. Sect. 241(d); 5 U.S.C. Sect. 552a; 44 U.S.C. Sect. 3501 et seq.

††† Fully alcohol-attributable conditions include the 100% alcohol-attributable chronic causes as well as the 100% alcohol-attributable acute causes (i.e., alcohol poisonings that are a subset of deaths in the alcohol-related poisonings category and suicide by exposure to alcohol that are a subset of deaths in the suicide category).

§§§
https://monitoringthefuture.org/wp-content/uploads/2023/07/mtfpanel2023.pdf

¶¶¶
https://alcoholpolicy.niaaa.nih.gov/sites/default/files/file-page/digest_state_alcohol_policies_in_response_to_covid-19_220101.pdf

****
https://www.cdc.gov/mmwr/volumes/70/wr/mm7015a3.htm

††††
https://onlinelibrary.wiley.com/doi/10.1111/joim.13545

§§§§
https://www.cdc.gov/alcohol/fact-sheets/prevention.htm

¶¶¶¶
https://www.cdc.gov/alcohol/checkyourdrinking/index.html

*****
https://www.uspreventiveservicestaskforce.org/uspstf/recommendation/unhealthy-alcohol-use-in-adolescents-and-adults-screening-and-behavioral-counseling-interventions

†††††
https://www.thecommunityguide.org/findings/health-communication-and-social-marketing-campaigns-include-mass-media-and-health-related.html
